# Nutraceutical Approach to Non-Alcoholic Fatty Liver Disease (NAFLD): The Available Clinical Evidence

**DOI:** 10.3390/nu10091153

**Published:** 2018-08-23

**Authors:** Arrigo F. G. Cicero, Alessandro Colletti, Stefano Bellentani

**Affiliations:** 1 Italian Society of Nutraceuticals (SINut), Medical and Surgical Science Department, University of Bologna, 40138 Bologna, Italy; dott.alessandro.colletti@gmail.com; 2 Gastroenterology and Hepatology Service-Clinica Santa Chiara, 6604 Locarno, Switzerland; bellentanistefano@gmail.com

**Keywords:** NAFLD, dietary supplements, nutraceuticals, clinical trials

## Abstract

Non-alcoholic fatty liver disease (NAFLD) is a clinical condition characterized by lipid infiltration of the liver, highly prevalent in the general population affecting 25% of adults, with a doubled prevalence in diabetic and obese patients. Almost 1/3 of NAFLD evolves in Non-Alcoholic SteatoHepatitis (NASH), and this can lead to fibrosis and cirrhosis of the liver. However, the main causes of mortality of patients with NAFLD are cardiovascular diseases. At present, there are no specific drugs approved on the market for the treatment of NAFLD, and the treatment is essentially based on optimization of lifestyle. However, some nutraceuticals could contribute to the improvement of lipid infiltration of the liver and of the related anthropometric, haemodynamic, and/or biochemical parameters. The aim of this paper is to review the available clinical data on the effect of nutraceuticals on NAFLD and NAFLD-related parameters. Relatively few nutraceutical molecules have been adequately studied for their effects on NAFLD. Among these, we have analysed in detail the effects of silymarin, vitamin E, vitamin D, polyunsaturated fatty acids of the omega-3 series, astaxanthin, coenzyme Q10, berberine, curcumin, resveratrol, extracts of *Salvia milthiorriza*, and probiotics. In conclusion, Silymarin, vitamin E and vitamin D, polyunsaturated fatty acids of the omega-3 series, coenzyme Q10, berberine and curcumin, if well dosed and administered for medium–long periods, and associated to lifestyle changes, could exert positive effects on NAFLD and NAFLD-related parameters.

## 1. Introduction

The liver is the largest gland in the human body. It has an antrophrenic action (both endocrine and exocrine) and more than 150 other functions. In particular, the liver interacts with the glyco-lipid metabolism, being responsible for gluconeogenesis, glycogenolysis, glycogenosynthesis, apolipoprotein synthesis, cholesterol, and triglycerides, and Low-Density Lipoprotein (LDL) cholesterol elimination through biliary route. The bile produced by the liver is also essential for emulsifying lipids in the intestine allowing absorption. The liver is also responsible for maintaining plasma osmolarity, through the production of albumin and globulins, for the production of coagulation factors, such as factor I (fibrinogen), II (thrombin), V, VII, IX, X, XI, and other proteins involved in coagulation processes, such as protein C, protein S, hepcidin, and antithrombin. In addition to these, it also produces other proteins and enzymes essential for survival (e.g., alpha-1 antitrypsin). The liver is also responsible for the catabolism of endogenous toxicants, for the storage of glycogen, vitamin B12, iron and copper, and it mainly contributes to the function of the immune system. For all these reasons, it is evident that liver health is essential for maintenance of the health of the entire organism and must be preserved as much as possible [1].

Intracellular infiltration of fat in the liver is called liver steatosis and could be due to both excessive alcohol intake (Alcoholic Fatty Liver Disease—AFLD) and other metabolic factors (Non-Alcoholic Fatty Liver Disease—NAFLD). The international threshold level chosen by the Scientific Community to distinguish AFLD from NAFLD is 2 drinks, the equivalent of 20 g, per day. NAFLD is an extremely common condition affecting 25–30% of the general adult population, 15% of children, and more than 50% of overweight, obese, and type 2 diabetics. NAFLD could not be considered a real disease even if absolutely reversible [2]. 

The clinically aggressive variant of NAFLD, non-alcoholic steatohepatitis (NASH), characterized by inflammation and progressive tissue degeneration, affects about 5% of the general adult population, and 20% of obese people [3]. The gold standard for NAFLD and NASH diagnosis is liver biopsy. However, the diagnosis of NAFLD is usually made by ultrasounds (“bright liver”), after excluding other causes of chronic liver disease, an alcohol intake lower of 20 g/day, and by using validated scores, such as the Fatty Liver Index (FLI), or fibrosis score or others [4]. 

The main risk factors for primary NAFL and NAFLD (Table 1) are overweight/obesity, insulin resistance/type 2 diabetes, hypertriglyceridaemia and the related dietary-behavioural triggers, primarily the intake of beverages sweetened with fructose. By itself, in different observational studies [5], only consumption of sugared soft drinks (mainly with fructose) increases the risk of developing NAFLD of around 55% [6]. Among the emerging risk factors there are also the smoking habit and the Obstructive Sleep Apnoea Syndrome (OSAS), but also insomnia and excessive daytime sleepiness unrelated to nocturnal sleep apnoea [7,8]. However, the first risk factor is often related to poor lifestyle habits. Finally, the strong association between hypothyroidism and NAFLD has recently been confirmed by a meta-analysis of 13 prospective studies that showed how hypothyroidism could increase the risk of NAFLD to more than 50%. The risk increases up to 70% if subclinical hypothyroidism is excluded [9].

NAFLD is the first step for the development of irreversible alterations of the liver parenchyma leading to cirrhosis (about 1/3 of the cases of NAFLD tend to become NASH, and 15% of these can degenerate into cirrhosis), while on the other hand, NAFLD is itself a risk factor for the development of cardiovascular disease [10] and type 2 diabetes, and preliminary data suggest that it may also be associated with a greater incidence of hepatic and extra-hepatic oncology pathologies [11]. A recent meta-analysis of 9 observational studies that included data from 96,595 adult subjects (34.1% of whom were affected by NAFLD) with 4654 cases of moderate-to-severe renal failure over a median observation period of 5.2 years showed a risk of also developing chronic renal failure 37% higher in patients with NAFLD. This risk was the greater the degree of lipid infiltration of the liver. Considering that both renal failure and NAFLD in themselves are then risk factors for cardiovascular disease, it is easy to understand how this epidemiological association is of particular relevance [12]. 

Another meta-analysis of six studies that included 25,837 patients (of whom 5953 were affected by NAFLD) showed that patients with NAFLD had a relative risk of total cardiovascular events of 1.77 (95% CI 1.26–2.48, *p* < 0.001). Specifically, the relative risk increased to 2.26 (95% CI: 1.04–4.92, *p* < 0.001) with regard to coronary artery disease and to 2.09 (95% CI: 1.46–2.98, *p* < 0.001) relative to ischemic stroke. Furthermore, the presence of NAFLD seems to significantly increase the relative risk of mortality due to cardiovascular causes (RR 1.46, 95% CI 1.31–1.64, *p* < 0.001) [13].

Pending specific drug therapies, actually in phase 3 clinical trials, the main treatment of NAFLD is currently the improvement of lifestyle aimed at weight loss and increased physical activities, in order to reduce insulin resistance [14]. Since the risk factors for NAFLD are similar to the ones for cardiovascular diseases, the suggestions for the management of NAFLD are the same suggested by the guidelines for the prevention of cardiovascular diseases [15]. Therefore, the general indication is to prescribe relatively low-calorie diets (with a caloric quantity proportional to energy consumption), with carbohydrates predominantly with a low glycemic index and minimizing the consumption of fructose, alcohol, saturated and trans-unsaturated fats [16]. In particular, the adherence to Mediterranean Diet is a significant predictor of changes in the fat content of the liver in patients with NAFLD [17]. The consumption of coffee will not be prescribed but if consumed by the patient it will not be suggested to quit it, since there are many evidences, summarised in a recent metanalysis, showing a lower risk of fibrosis in coffee drinkers with NAFLD [18].

The role of physical activity is fundamental, to be implemented as much as possible according to the degree of training and any co-morbidities. In fact, a recent meta-analysis of 6 cohort studies involving 142,781 participants with 32,657 incident cases of NAFLD, as well as 4 case-control studies involving 382 affected patients and 302 controls, shows that the difference in risk of developing NAFLD among sedentary and physically active is 21% in observational studies and 57% in case-control studies [19]. On the other hand, regardless of diet, the greater the frequency and intensity of physical activity, the greater the reduction of transaminase levels and the degree of hepatosteatosis, especially in overweight subjects [20].

To this dutiful approach, increasing evidence suggests that the prescription of specific supplements or nutraceuticals with demonstrated hepatoprotective action can be suggested to accelerate the improvement of the alteration of liver enzymes and maybe of liver steatosis or at least to slow down its evolution [9]. This rationale becomes stronger when using nutraceuticals simultaneously active on the modulation of cardiovascular risk.

## 2. Methods

For the purpose of this review, a systematic search strategy was developed to identify trials in both MEDLINE (National Library of Medicine, Bethesda, Maryland, MD, USA; January 1980 to May 2018) and the Cochrane Register of Controlled Trials (The Cochrane Collaboration, Oxford, UK). The terms ‘nutraceuticals’, ‘dietary supplements’, ‘food bioactives’, ‘herbal drug’, ‘liver steatosis’, ‘non-alcoholic fatty liver disease’, ‘NAFLD’, ‘clinical trial’, and ‘human’ were incorporated into an electronic search strategy. The references of all identified studies and review articles were reviewed to look for additional studies of interest. The authors reviewed all of the citations retrieved from the electronic search to identify potentially relevant articles for this review. We excluded in vitro data and animal studies and focused on human data, in order to limit our report to food components and nutraceuticals for which safety and tolerability in humans are already known. Therefore, we preferably selected papers reporting recent comprehensive reviews or meta-analyses, or original clinical trials on substances with a lipid-lowering effect and those improving action on vascular health. 

## 3. Results

From a “nutraceutical point of view”, there are relatively few molecules adequately clinically studied for their effects on NAFLD: among these, we will analyse in detail the role of silymarin, vitamin E, vitamin D, polyunsaturated fatty acids of the omega-3 series, astaxanthin, coenzyme Q10, berberine, curcumin, resveratrol, extracts of *Salvia milthiorriza*, and probiotics.

### 3.1. Silymarin

**Figure 1:**
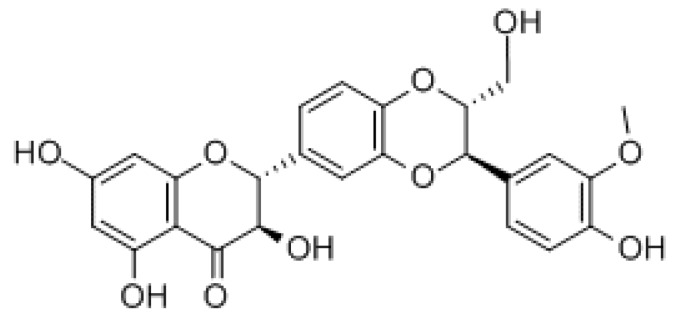
Chemical structure of silybin.

Silymarin is a powerful antioxidant agent extracted from milk thistle (*Silybum marianum*) with a specific liver tropism. Silymarin is actually a set of antioxidant substances of which the most concentrated are six flavolignans (silibine A and B—usually in isoconcentration—, isosilibine, silidianin, silicristine, isosilicristine) and a flavonoid (taxifolin) [21]. Of these substances, the one with the highest concentration and the most evident biological effects is silybin (Figure 1), (ID IUPAC: (2R, 3R) -3,5,7-trihydroxy-2-(2R, 3R)-3-(4-hydroxy-3-methoxyphenyl)-2-(hydroxymethyl) -2,3-dihydrobenzo [b] [1,4] dioxin-6-yl] chroman-4-one, molecular mass 482,44 g/mol), constituting up to 70% of silymarin in its diastereoisomeric silybin A and silybin B forms [22].

Silymarin has poor oral bioavailability, both due to poor intestinal absorption and high hepatic first-pass metabolism. However, this limitation can be bypassed with particular pharmaceutical techniques, for example by complexing silymarin in a phytosome with phosphatidylcholine, which increases its solubility while maintaining its antioxidant properties [23]. Silymarin is undoubtedly one of the most studied nutraceuticals of plant origin in hepatopathic patients, even with severe disease [24]. The pharmacological mechanisms by which silymarin exerts its hepatoprotective action in patients with NAFLD are numerous and summarized in Table 2 [25], which also highlights those that could have a positive impact on vascular health.

The available data show the ability of silymarin (administered alone, or usually in combination with low dosages of vitamin E) to improve insulin resistance and indirect markers of hepatosteatosis (Hepatic Steatosis Index, Lipid Accumulation Product) already after 3 months of treatment [26].

In a recent multicenter randomized double-blind phase 3 study conducted on 180 patients with histological diagnosis of NAFLD/NASH, the administration of silybin and vitamin E (silibine 188 mg, phosphatidylcholine 388 mg, vitamin E 180 mg) for 12 months determined the normalisation of transaminase, a significant reduction of gamma-glutamyl transferase levels and the significant decrease of liver steatosis measured with both ultrasound scan and, in one-fifth of patients, with a second liver biopsy. As expected, there was also a proportional improvement of fasting glucose, basal insulinemia and insulin resistance index [27]. These data were confirmed by a meta-analysis of 8 controlled clinical trials involving 587 patients [28]. There is also a preliminary report showing that supplementation with 420 mg/day of silymarin reduced the 4-years risk of mortality in patients with cirrhosis [29]. Overall tolerability is usually good, even for high doses and long-term administration [30]. For these reasons, the guidelines of the Mayo Clinic on food supplements classifies the use of silymarin for hepatoprotection as Grade B (“Good scientific evidence for this use”) [31].

Of particular interest from a cardiometabolic point of view is the clinical effect of silymarin on the metabolic control of the diabetic patient. A recent meta-analysis of five controlled clinical trials that enrolled 270 patients showed how the administration of silymarin significantly improved fasting glycemic control (−26.86 mg/dL; 95% CI −35.42–18.30) and glycated hemoglobin values (−1.07; 95% CI −1.73–0.40), plausibly for the insulin-sensitizing action of this nutraceutical [32].

The greatest limitation in the use of silymarin is usually the cost of effective dose treatment, which needs to be administered continuously and protracted over time at least until optimisation of the lifestyle, and normalisation of ultrasound liver scan.

### 3.2. Vitamin E

**Figure 2:**
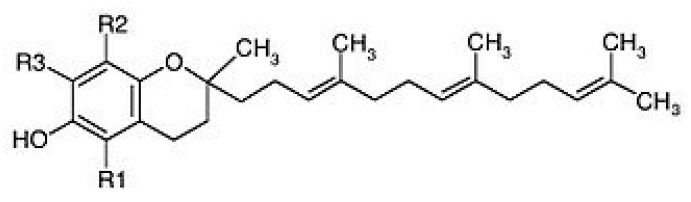
Chemical structure of tocotrienols.

A nutraceutical tested extensively in patients with NAFLD, although almost always in association with silymarin, is vitamin E (alpha-tocopherol or (2*R*)-2,5,7,8-Tetramethyl-2-[(4*R*,8*R*)-(4,8,12-trimethyltridecyl)]chroman-6-ol) (Figure 2).

The most effective dosage of the active form of Vitamin E in order to reduce inflammation and liver fibrosis is 40 times higher than the Recommended Daily Allowance (RDA) (800 IU/day). However, doses 20 times higher (400 IU/day) are associated with increased risk of mortality from all causes. Therefore, caution is necessary to set up long-term therapies with vitamin E at the effective dosage. The majority of clinicians use Vitamin E either at a lower, less-effective, but safer dosages or by associating it with other active drugs that are probably effective in the management of NAFLD [33]. A recent meta-analysis of 16 controlled clinical trials has shown that the long-term administration of low-dose Vitamin E and alone (not in association with other antioxidant vitamins) is able to reduce the risk of myocardial infarction (RR 0.82; 95%CI, 0.70–0.96; *p* = 0.01) [34]. Furthermore, another meta-analysis involving 303 subjects enrolled in seven studies showed that vitamin E supplementation is associated with a 2.5% increase in flow-mediated vasodilatation [35]. This result is important since it has been estimated that a 1% improvement in flow-mediated vasodilatation would be associated with a 12% reduction in cardiovascular risk [36].

### 3.3. Vitamin D

**Figure 3:**
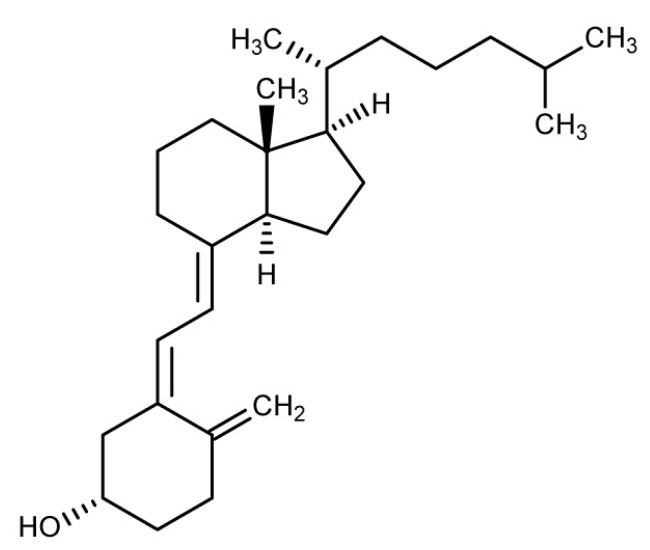
Chemical structure of Vitamin D3.

Vitamin D3 (1,25 OH cholecalciferol; C27H44O; ID IUPAC: (3β, 5Z, 7E)-9,10-secocholesta-5,7,10 (19)-trien-3-ol) (Figure 3) is a secosteroid hormone with a molar mass of 384.64 g/mol which plays a fundamental role in mineral metabolism, but also in the regulation of immune response, cell differentiation and inflammation, with important repercussions on both liver and cardiovascular health. In the human body, vitamin D is derived for approximately 10% from the diet, while from 90% from the cutaneous conversion of 7-dehydrocholesterol to colecalciferol for exposure to ultraviolet B (UVB). Then the cholecalciferol is hydroxylated from the liver by a 25-hydroxylase and then from the kidney from an alpha-1 hydroxylase, resulting in activation of 1,25-hydroxy-cholecalciferol or calcitriol vitamin D. The decline in exposure to the sun linked to the modern lifestyle, associated with the reduction in age of the capacity of hepatic and renal hydroxylation, make a large part of the population relatively and absolutely deficient in vitamin D [37].

Vitamin D deficiency and non-alcoholic hepatosteatosis could be associated only for the high prevalence of both conditions in the general population. However, recent epidemiological evidence shows that in patients with NAFLD they are more frequently deficient in vitamin D than in the general population, and the circulating vitamin D levels are proportional to the degree of fibrotic evolution of NAFLD [38]. Nevertheless, not all the studies are in agreement: a recent metanalysis of observational studies involving 974 NAFLD patients, showed no difference in 25-hydroxyvitamin D levels among NAFLD patients with high NAFLD activity score (NAS) versus low NAS (MD = −0.93, 95% CI −2.45 to 0.58) and also high fibrosis score versus low fibrosis score (MD = 0.88, 95% CI −2.65 to 4.42). Despite evidence implicating vitamin D in NAFLD pathogenesis, serum 25-hydroxyvitamin D may not be associated with NAFLD histologic severity [39]. In addition, in the study of Barchetta et al. oral vitamin D supplementation (2000 IU/day) over 24 weeks did not improve hepatic steatosis or metabolic/cardiovascular parameters in diabetic patients with NAFLD [40]. In contrast, the study of Lim et al. suggests that serum 25-hydroxyvitamin D levels may be a risk factor for metabolic syndrome in patients with NAFLD [41]. 

Table 3 summarizes the available evidence that binds vitamin D and NAFLD from a pathophysiological point of view, justifying vitamin D supplementation in patients affected by this condition [42,43]. Moreover, integration is justified by the virtual absence of side effects for non-pharmacological dosages of supplementation, because vitamin D deficiency is almost pandemic, and for the positive actions that vitamin performs not only at the level of the bone and liver, but also on the immune and cardiovascular system [44,45].

Some clinical studies show that daily vitamin D supplementation improves insulin resistance and related parameters in patients with NAFLD [54,55]. Again, vitamin D supplementation can also have positive implications on the cardiovascular system. In fact, while its deficiency has been associated with a risk of hypertension and vascular ageing. Its supplementation, on the contrary, would significantly reduce the levels of high sensitivity C reactive protein, known as an independent risk factor for cardiovascular diseases, as demonstrated by the meta-analysis of 10 studies involving 924 participants [56].

### 3.4. Polyunsaturated Fatty Acids of the Omega-3 Series

**Figure 4:**
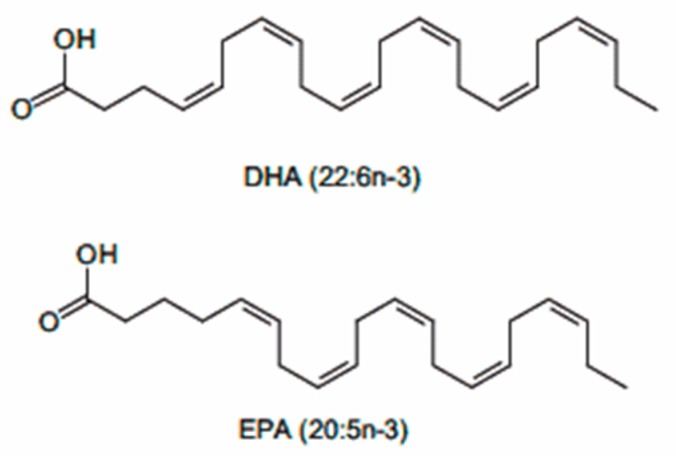
Chemical structure of Docosahexaenoic acid (DHA) and eicosapentaenoic acid (EPA).

The polyunsaturated fatty acids of the series omega-3 (Figure 4) are essential fatty acids that the human body is unable to synthesize and must take them with the diet.

In the human organism, omega-3 fatty acids are involved in a very high number of biological activities that make them fundamental for adequate development and for the maintenance of the health status of different organs and tissues. Although a balanced diet can theoretically provide an adequate amount of omega-3 fatty acids, the qualitative and biodiversity impoverishment of the components of the diet, the methods of processing and cooking, and the increase in functional demands of the organism makes supplementation with omega-3 fatty acids increasingly necessary [57]. A recent meta-analysis of controlled clinical trials has shown that supplementation with polyunsaturated fatty acids of the omega-3 series, mainly docosahexaenoic acid (DHA), and eicosapentaenoic acid (EPA), contributes significantly to the reduction of circulating levels of AST and gamma-glutamyl transferase [58]. This effect, associated with the known hypotriglyceridemic and anti-inflammatory actions of omega-3 fatty acids, places them among the potentially active and effective nutraceuticals in the management of NAFLD and NASH [59]. A meta-analysis of 4 randomized clinical trials conducted in 263 children showed that long-term supplementation with EPA and DHA is associated with a 25% reduction of both circulating levels of AST and ALT, and the degree of steatosis assessed by liver ultrasound scan, with no side effects [60].

### 3.5. Astaxanthin

**Figure 5:**
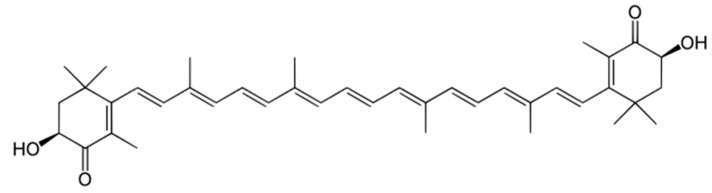
Chemical structure of Astaxanthin.

A possible nutraceutical alternative to vitamin E (to be considered to avoid the problem of balancing between hepatoprotective dose and cardio-injurious dose) is the use of astaxanthin (C40H52O4: ID IUPAC: (6S)-6-Hydroxy-3-[(1E,3E,5E,7E,9E,11E,13E,15E,17E)-18-[(4S)-4-hydroxy-2,6,6-trimethyl-3-oxo-1-cyclohexenyl]-3,7,12,16-tetramethyloctadeca-1,3,5,7,9,11,13,15,17-nonaenyl]-2,4,4-trimethyl-1-cyclohex-2-enone), a carotenoid of marine origin of the colour purplish-red, very stable, with an antioxidant activity in vitro extremely more powerful than the most common natural antioxidants (e.g., vitamins A, E, and C, lycopene, resveratrol), with a molar mass of 596,841 g/mol (Figure 5) [61]. In preclinical experimental models, astaxanthin is more effective than vitamin E in reducing lipogenesis, insulin resistance, hepatic inflammation, and fibrogenesis, and therefore appears to be the ideal natural antioxidant for the prevention of liver injury induced by NAFLD [62]. However, there is still no direct evidence on humans of these promising data.

### 3.6. Coenzyme Q10

**Figure 6:**
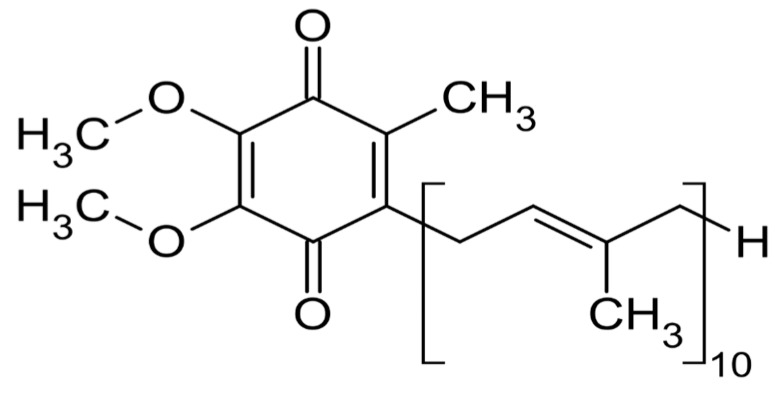
Chemical structure of coenzyme Q10.

Another antioxidant of recent interest for the management of NAFLD is Coenzyme Q10 (Ubidecarenone; C59H90O4; ID IUPAC: 2-[(2E, 6E, 10E, 14E, 18E, 22E, 26E, 30E, 34E)-3.7, 11,15,19,23,27,31,35,39-Decamethyltetraconta-2,6,10,14,18,22,26,30,34,38-decaenyl]-5,6-dimethoxy-3-methylcyclohexa-2,5-diene-1,4-dione), molecule of molar mass 863.34 g/mol, particularly concentrated in heart, striated muscle and liver, but present in all cells of the organism (Figure 6) [63]. 

In a recent double-blind randomized placebo clinical study, 100 mg/day of Coenzyme Q10 for 3 weeks resulted in a significant reduction of transaminases, gamma-GT, hsCRP and degrees of NAFLD, as well as improvement of the adiponectin/leptin ratio [64]. In addition, coenzyme Q10 could help to improve the lipid pattern typically associated with NAFLD [65], as well as reducing oxidized LDL levels and arterial pressure. The main limitation of the use of coenzyme Q10 is the need for high dosages (>100 mg/day) of pharmaceutically modified formulations to have an increased bioavailability, since coenzyme Q10 by itself has poor oral bioavailability. On the other hand, it has a very high safety profile without any risk of drug interactions [66].

### 3.7. Berberine

**Figure 7:**
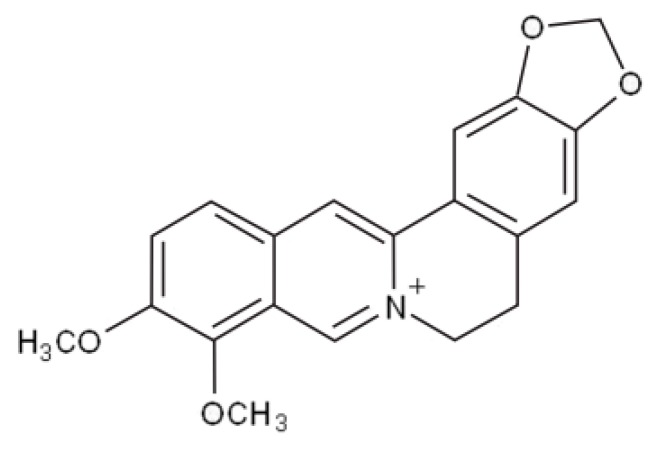
Chemical structure of berberine.

Berberine hydrochloride is a quaternary ammonium salt belonging to the group of alkaloids benzylisoquinoline (C20H18NO4 +) of a molar mass 336.3612 g/mol (Figure 7), extracted from numerous medicinal plants (in particular those of the *Berberis* genus) and endowed with lipid-lowering and insulin-sensitizing actions clearly demonstrated in humans [67]. 

Some preliminary clinical reports confirmed that these actions of berberine are also related to the improvement of levels of indirect markers of hepatosteatosis (Hepatic Steatosis Index, Lipid Accumulation Product) for short-term supplements (2–4 months) at doses of 500 mg/day [68]. These data were recently collected in a meta-analysis of six randomized clinical trials that evaluated 501 patients, confirming the positive effect of berberine on lipid parameters, insulin resistance, hepatic markers and degree of hepatic steatosis in patients with NAFLD. However, these studies have used relatively high doses (1000–1500 mg/day), which may be associated with intestinal disorders [69]. Even berberine has a poor oral bioavailability that can, however, be improved by ad hoc pharmaceutical techniques.

### 3.8. Curcumin

**Figure 8:**
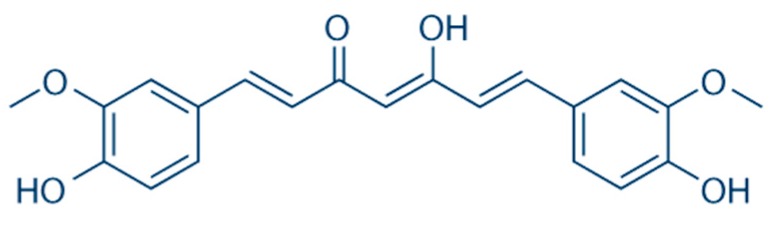
Chemical structure of curcumin.

Curcumin (C21H20O6, ID IUPAC: (1E, 6E)-1,7-bis (4-hydroxy-3-methoxyphenyl)-1,6-heptadiene-3,5-dione, molar mass: 368.38 g/mol) (Figure 8), another known insulin-sensitizing agent extracted from *Curcuma longa*, has been associated in numerous preclinical studies and in recent preliminary clinical trials with a significant improvement in NAFLD-related parameters [70]. In particular, a clinical study of 100 Asian patients with metabolic syndrome has shown a statistically significant improvement in the degree of hepatic steatosis assessed by liver ultrasound with a daily administration of 400 mg of curcumin [71]. In another controlled clinical study conducted on 102 Iranian patients, supplementation with phytosomal curcumin, 500 mg b.i.d. for 8 weeks led to a significant reduction in transaminase levels, waist circumference, body mass index, but especially the degree of hepatic steatosis in 75% of the treated subjects. Although these data are particularly promising, it should be noted that the positive effects are often observed for high supplementations (usually >1500 mg/day) of pure curcumin, with consequent problems of treatment compliance and costs (partially mitigated with the use of new, more bioavailable pharmaceutical formulations) [72]. This is because curcumin also has a poor oral bioavailability, that can, however, be improved by “ad hoc” pharmaceutical techniques.

### 3.9. Resveratrol

**Figure 9:**
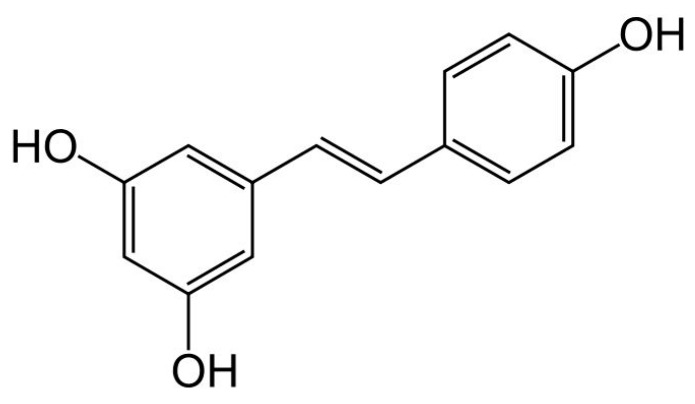
Chemical structure of resveratrol.

Resveratrol (3,5,4′-trihydroxy-trans-stilbene, C14H12O3, molar mass: 228.25 g/mol) is a non-flavonoid phenol (Figure 9) particularly concentrated in the grape skin peel, but also in the extracts of *Polygonum cuspidatum*, poorly orally bioavailable (if not chemically modified), characterized by an important antioxidant, vasoprotective (both cerebral and peripheral) and insulin-sensitizing activity [73,74]. Preliminary clinical data appear to contradict preclinical literature, suggesting a potential efficacy of resveratrol in improving NAFLD-related parameters [75]. However, these studies are usually short (too short to have an impact on the liver structure) and are conducted with resveratrol doses incompatible with the insulin-sensitizing action that could improve NAFLD. In fact, when used at the appropriate dose and for long periods, resveratrol has already shown to exert an important antihypertensive effect in those affected by NAFLD [76].

### 3.10. Salvia Miltiorrhiza 

A meta-analysis of 8 controlled clinical trials that included 800 Asian patients showed how *Salvia miltiorrhiza* dry extract supplementation (red or Chinese sage, Danshen) leads to a significant reduction in plasma levels of transaminases associated with an increase in computerized tomographic contrast between liver and spleen, indicative of a reduction in the degree of hepatosteatosis [77]. Despite the interest in these evidences, clinical data on Caucasian subjects are currently lacking.

### 3.11. Probiotics

Growing literature shows that even chronic supplementation with probiotics and/or symbiotics could improve several parameters related to NAFLD (insulin resistance, plasma levels of transaminases, degree of lipid infiltration of the liver), but the available studies have all been conducted with different probiotic strains or associations and it is therefore difficult to provide a specific indication for supplementation [78]. Undoubtedly, if probiotics are to be taken for other indications, the effect on the possibly co-hosted NAFLD could only be positive [79].

Promising results may come from certain probiotic strains. Preliminary RCTs have shown that treatment with *L. bulgaris* or *S. thermophilus* reduces the levels of alanine aminotransferase, aspartate aminotransferase, and gamma glutamyltransferase [80]. In obese children with NAFLD, supplementation with *L. rhamnosus* GG resulted in a significant improvement in liver function [81]. Alisi et al. found a significant improvement in the severity of fatty liver and a significant decrease in Body Mass Index (BMI) of children with NAFLD treated for 4 months with bifidobacteria, lactobacilli and *S. thermophilus* strains. These data suggest that probiotics could reduce liver fat and thus prevent the progression of NAFLD [82]. Similar results were obtained with the association *L. acidophilus* and *B. lactis* for 8 weeks in adult patients with NAFLD: at the end of treatment, there was a significant improvement of transaminases and cholesterolaemia compared to the control group [83].

## 4. Discussion

At the state of the art, in the absence of specific pharmacological therapies available, some nutraceuticals could play an important role in improving the NAFLD frameworks in association with diet and lifestyle based on weight loss and the reduction of insulin resistance. In particular, data concerning silymarin, vitamin E polyunsaturated fatty acids of the omega-3 series, coenzyme Q10, berberine and curcumin (Table 4), all nutraceuticals with both hepatoprotective activity and positive actions on the cardiovascular system, seem particularly convincing. Results of vitamin D still remain conflicting. As regards herbal extract, the clinical literature often does not report details about the extracts standardization.

Many other nutraceuticals could exert some positive effects on NAFLD. For instance, L-carnitine might be an interesting option as adjuvant in people with NAFLD for its role in a number of intracellular and metabolic functions [85]: in preliminary clinical studies, it seems to improve insulin resistance and inflammatory biomarkers, even if data from an RCT of supplementation with 500 mg/twice daily of this molecule for 1 year for the treatment for NAFLD showed no significant changes in liver function tests and ultrasound grade [86].

The role of flaxseed in NAFLD was recently assessed in a RCT. In a total of 50 people with NAFLD (confirmed by fibroscan examination) the supplementation with 30 g/day of brown milled flaxseed for 12 weeks was associated to an improvement in BMI, waist circumference, serum transaminases, hs-CRP, TNF-alpha, glucose and insulin concentrations compared to control, while HOMA-IR, hepatic fibrosis and steatosis scores were improved in both groups even if significantly greater in the flaxseed group compared to control [87].

*Phyllanthus urinaria* has been shown to reduce hepatic steatosis and necroinflammation in vitro and in vivo [88]. In a RCT of 60 patients with histology-confirmed NASH treated with 1 g of *P. urinaria* three times daily or placebo for 24 weeks, the NAFLD activity score, steatosis percentage, and steatosis grade assessed by biopsy were significantly reduced from baseline in the intervention group, while changes were not significant in the control group [89]. The bioactive compound responsible for this effect is not fully clarified.

Anthocyanins, polyphenols other than resveratrol and betaine, are compounds with preliminary data regarding, in particular, the improvement of hyperlipidaemia and the reduction of oxidative stress and vascular inflammation: however, studies on people with liver steatosis are still lacking [90]. Again, clinical data for garlic, *Chlorella vulgaris*, Myrica (bayberry) and green tea are few and in general concern short-term RCTs [91].

Finally, many traditional Chinese herbal formulas are reported in literature to have significant anti-NAFLD effects: the association of *Artemisia capillaris* (Thunb), *Gardenia jasminoides* (Ellis), and *Rheum palmatum* (L) can reduce the accumulation of hepatic fat, increase endothelial progenitor cell proliferation, enhance adiponectin secretion, and increase PPAR-γ expression [92].

In conclusion, a relatively large number of dietary supplements and herbal extracts seems to improve NAFLD and related parameters. However, long-term double-blind randomised clinical trials are still needed to understand if the observed results are confirmed and maintained in time. Furthermore, the molecular targets and the signalling transduction pathways of many of these nutraceuticals should still be more deeply investigated. Particularly, in plant extracts, the eventual additive or synergistic effect of each single bioactive compounds must be clarified.

## Figures and Tables

**Table 1:** Main risk factors for the development of Non-Alcoholic Fatty Liver Disease (NAFLD).

Defined	Emerging
Diet rich in refined foods, carbohydrates with a high glycemic index, drinks sweetened with fructose Sedentary Overweight/Obesity Insulin resistance/Type II diabetes Intake of cortisones, methotrexate, some antiretrovirals	Sarcopenia Hypothyroidism Hyperuricemia Cigarette smoke Chronic obstructive pulmonary disease (COPD) Polycystic ovary syndrome *Helicobacter pylori* infection

**Table 2:** Biological effects of silymarin involved in its hepatoprotective action in patients with NAFLD.

Effect	Proposed Mechanism of Action
**Antioxidant**	Direct scavenger activity * Mitochondrial function optimization * Activation of protective molecules such as Heat Shock Proteins, thioredoxin and sirtuins
**Anti-inflammatory**	Inhibition of NF-κB activity Proinflammatory cytokine synthesis reduction (IL-1, IL-6, TNF-α, TNF-β) *
**Anti-apoptotic**	Modulation of caspase release and TNF-α effect
**Antifibrotic**	Inhibition of the conversion of stellate cells into fibroblasts Downregulation of the expression of profibrotic genes (procollagen III, TGF-β)
**Endocrine-metabolic**	Partial activation of estrogen receptors * Insulin-sensitizing action * PPAR-agonist action * Increased expression of GLUT4 on the cell surface * Inhibition of Hydroxy-Methyl-Glutaryl Coenzyme A reductase *
**Choleretic**	Upregulation of the bile salt export pump *

GLUT4 = glucose transporter type 4, IL = Interleukin, NF-κB = nuclear factor kappa-light-chain-enhancer of activated B cells, PPAR = peroxisome proliferator-activated receptor, TGF-β = transforming growth factor beta, TNF = tumor necrosis factor, * potentially positive effects on vascular health.

**Table 3:** Pathophysiological Mechanisms That Bind Vitamin D and NAFLD.

Proposed Mechanism	Support Tests	Ref.
**Insulin-sensitivity improvement**	Mice lacking vitamin D receptors are insulin-resistant Vitamin D modulates the transcription of the insulin gene Vitamin D deficiency worsens the secretory response of beta-cells in response to carbohydrate loading Vitamin D improves glucose transport in muscle cells Vitamin D upregulates the translocation of GLUT4 and the use of glucose by adipocytes	[46,47]
**Reduction of inflammation of adipose tissue**	Higher levels of liver vitamin D are associated with higher levels of adiponectin (inversely proportional to adipocytic flogosis) In animal models, vitamin D supplementation reduces the amount of IL-6 in adipocytes Treatment of human adipocytes with vitamin D inhibits NF-kB and reduces the release of proinflammatory cytokines Vitamin D inhibits the chemotaxis of macrophages and increases the expression of adiponectin in preadipocytes	[48,49,50]
**Reduction of hepatic inflammation**	Vitamin D deficiency triggers Toll receptors and exacerbates liver inflammation Artificial lighting in rats reduces the degree of inflammation and hepatic apoptosis The expression of the vitamin D receptor on cholangiocytes is inversely proportional to the severity of steatosis and NAFLD scores	[51,52]
**Slowdown of liver fibrosis**	Vitamin D inhibits the proliferation of hepatic stellate cells in vitro Vitamin D reduces pro-fibrotic marker levels (as TIMP-1) and the production of type I collagen in cell cultures of hepatic stellate cells Vitamin D receptor knockout mice spontaneously develop hepatic fibrosis	[53]

GLUT4 = glucose transporter type 4, IL = Interleukin, NF-kB = nuclear factor kappa-light-chain-enhancer of activated B cells, TIMP-1 = tissue inhibitors of metalloproteinases-1.

**Table 4:** Nutraceuticals with clinical effects on NAFLD: Main mechanisms of action, clinical effects, tested dosages, side effects, and level of clinical Evidence.

Nutraceutical	Tested Dosages	Proposed Mechanism of Actions	Clinical Effects	Side Effects	Level of Scientific Evidence [Ref.]
**Berberine**	500–500 mg/day	Activation of AMPK and the expression of LDL receptors, inhibition of PCSK9	Improvement of levels of indirect markers of hepatosteatosis (Hepatic Steatosis Index, Lipid Accumulation Product), lipid parameters and insulin resistance	Mild gastrointestinal side effects	Meta-analysis of RCTs [69,84]
**Coenzyme Q10**	100–300 mg/day	Antioxidant activity, sensitizing of Ca++ channels, inductor of the synthesis of ATP, reduction of oxidative stress and lipid peroxidation	Reduction of transaminases, gamma-GT, hsCRP and degrees of NAFLD and hepatic steatosis, improvement of the adiponectin/leptin ratio	Not reported	RCTs [64,65,68]
**Curcumin**	400–2000 mg/day	Inhibition of the expression of NPC1L1 transporter, increases the efflux of cholesterol, downregulation of the expression of PCSK9, reduction of TNF-α levels, inhibition of NF-κB activation, lipid peroxidation and lysosomal enzyme activities, induction of PPAR-γ and Nrf2 activation	Improvement in the degree of hepatic steatosis, reduction in transaminase levels, waist circumference and body mass index	Mild nausea, stomach cramps and/or upset, diarrhea, dizziness	Meta-analysis of RCTs [71,72]
**Polyunsaturated Omega-3 fatty acids**	1–4 g/day of eicosapentanoic and/or docosahexaenoic acid	Reduction of the release and synthesis of inflammatory cytokines, activation of eNOS, prostaglandins synthesis balance toward vasodilating ones, insulin-sensitivity, vascular tone regulation by parasympathetic nervous system stimulation, and suppression of the renin–angiotensin–aldosterone system	Reduction of transaminases, serum triglycerides, blood pressure (SBP 1–5 mmHg)	Mild aftertaste, nausea, gastroesophageal reflux, bloating and dyspepsia	Meta-analysis of RCTs [58,59]
**Probiotics**	>3,5 CFU/day (extremely variable depending on strains, associations, and vehicle of administration used)	Reduction of lucky gut syndrome, intestinal permeability, modulation of bile salt hydrolases	Improvement of insulin resistance, plasma levels of transaminases, degree of lipid infiltration of the liver	Not reported	RCTs [78]
**Resveratrol**	>150 mg/day	Antioxidant, vasoprotective (both cerebral and peripheral) and insulin-sensitizing activity	Unclear	Rare gastrointestinal side effects	Open-label clinical studies [75,76]
**Silymarin**	150–450 mg/day	Direct scavenger activity, mitochondrial function optimization, activation of protective molecules such as HSPs, thioredoxin and sirtuins, inhibition of NF-kB activity, proinflammatory cytokine synthesis reduction (IL-1, IL-6, TNF-α), modulation of caspase release and TNF-α effect, inhibition of the conversion of stellate cells into fibroblasts, downregulation of the expression of profibrotic genes (procollagen III, TGF-β), partial activation of estrogen receptors, insulin-sensitizing action, PPAR-agonist action, increased expression of GLUT4 on the cell surface, inhibition of HMG-CoA reductase, upregulation of the bile salt export pump	Transaminase normalization, reduction of gamma-glutamyl transferase levels and degree of ultrasound-related liver steatosis, improvement of fasting glucose, basal insulinemia and insulin resistance	Mild gastrointestinal side effects	Meta-analysis of RCTs [29,30,31]
**Vitamin D**	2000–50,000 UI/day	Upregulation of the translocation of GLUT4, modulation of transcription of insulin gene, inhibition of NF-kB, release of proinflammatory cytokines and proliferation of hepatic stellate cells	Improvement of insulin sensitivity, hepatic and adipose inflammation	Not reported	RCTs [54,55]
**Vitamin E**	800 UI/day	Antioxidant	Improvement of arterial stiffness and reduction of risk of myocardial infarction	At 400 UI/day: increases risk of mortality (?)	Meta-analysis of RCTs [33,34,35]

AMPK = Adenosin-Monophosphate-Kinase-alpha, CFU = colony forming units, eNOS = endothelial nitric oxide synthase, GLUT4 = glucose transporter type 4, HMG-CoA = Hydroxy-Methyl-Glutaryl Coenzyme A, hs-CRP = high sensible C-reactive protein, IL = Interleukin, NF-κB = nuclear factor kappa-light-chain-enhancer of activated B cells, NPC1L1 = Niemann-Pick C1-Like 1) Nrf2 = nuclear factor erythroid-2-related factor-2, PCSK9 = proprotein convertase subtilisin/kexin type 9, PPAR = peroxisome proliferator-activated receptor, TGF-β = transforming growth factor beta, TNF = tumor necrosis factor, RCTs = Randomized clinical trials.
